# Mobile App Intervention to Reduce Substance Use, Gambling, and Digital Media Use in Vocational School Students: Exploratory Analysis of the Intervention Arm of a Randomized Controlled Trial

**DOI:** 10.2196/51307

**Published:** 2024-07-23

**Authors:** Kristin Grahlher, Matthis Morgenstern, Benjamin Pietsch, Elena Gomes de Matos, Monika Rossa, Kirsten Lochbühler, Anne Daubmann, Rainer Thomasius, Nicolas Arnaud

**Affiliations:** 1 German Centre for Addiction Research in Childhood and Adolescence University Medical Centre Hamburg-Eppendorf Hamburg Germany; 2 Institute for Therapy and Health Research IFT-Nord Kiel Germany; 3 IFT Institut für Therapieforschung Munich Germany; 4 Institute for Clinical Psychology and Psychotherapy Charlotte Fresenius University Munich Germany; 5 Institute for Medical Biometry and Epidemiology University Medical Centre Hamburg-Eppendorf Hamburg Germany

**Keywords:** prevention, vocational students, adolescents, mobile intervention, voluntary commitment, substance use, internet-related problems, mobile phone, adolescent, youths, student, students, use

## Abstract

**Background:**

During adolescence, substance use and digital media exposure usually peak and can become major health risks. Prevention activities are mainly implemented in the regular school setting, and youth outside this system are not reached. A mobile app (“Meine Zeit ohne”) has been developed specifically for vocational students and encourages participants to voluntarily reduce or abstain from a self-chosen addictive behavior including the use of a substance, gambling, or a media-related habit such as gaming or social media use for 2 weeks. Results from a randomized study indicate a significant impact on health-promoting behavior change after using the app. This exploratory study focuses on the intervention arm of this study, focusing on acceptance and differential effectiveness.

**Objective:**

The aims of this study were (1) to examine the characteristics of participants who used the app, (2) to explore the effectiveness of the mobile intervention depending on how the app was used and depending on participants’ characteristics, and (3) to study how variations in app use were related to participants’ baseline characteristics.

**Methods:**

Log data from study participants in the intervention group were analyzed including the frequency of app use (in days), selection of a specific challenge, and personal relevance (ie, the user was above a predefined risk score for a certain addictive behavior) of challenge selection (“congruent use”: eg, a smoker selected a challenge related to reducing or quitting smoking). Dichotomous outcomes (change vs no change) referred to past-month substance use, gambling, and media-related behaviors. The relationship between these variables was analyzed using binary, multilevel, mixed-effects logistic regression models.

**Results:**

The intervention group consisted of 2367 vocational students, and 1458 (61.6%; mean age 19.0, SD 3.5 years; 830/1458, 56.9% male) of them provided full data. Of these 1458 students, 894 (61.3%) started a challenge and could be included in the analysis (mean 18.7, SD 3.5 years; 363/894, 40.6% female). Of these 894 students, 466 (52.1%) were considered frequent app users with more than 4 days of active use over the 2-week period. The challenge area most often chosen in the analyzed sample was related to social media use (332/894, 37.1%). A total of 407 (45.5%) of the 894 students selected a challenge in a behavioral domain of personal relevance. The effects of app use on outcomes were higher when the area of individual challenge choice was equal to the area of behavior change, challenge choice was related to a behavior of personal relevance, and the individual risk of engaging in different addictive behaviors was high.

**Conclusions:**

The domain-specific effectiveness of the program was confirmed with no spillover between behavioral domains. Effectiveness appeared to be dependent on app use and users’ characteristics.

**Trial Registration:**

German Clinical Trials Register DRKS00023788; https://tinyurl.com/4pzpjkmj

**International Registered Report Identifier (IRRID):**

RR2-10.1186/s13063-022-06231-x

## Introduction

### Prevalence and Prevention of Substance Use in Vocational Schools

Substance use and other addictive behaviors such as gambling, screen-related gaming, and excessive use of digital media are widespread among adolescents and young adults across different cultures [1]. In Germany, the prevalence of substance-related and web-related addictive disorders peaks during adolescence [2-5]. Research suggests that vocational students are particularly vulnerable to substance use and addictive behaviors compared with age-matched peers in the general population [6].

Schools are an important setting for facilitating prevention programs because a large number of young people can be reached simultaneously [7]. However, vocational schools have been largely neglected concerning empirically evaluated prevention activities in Germany [8]. Approximately 50% of all graduates from conventional schools in Germany enter vocational training [9] and are thus not reached by available programs during the critical time of adolescence. This seems to be a serious limitation for national public health goals. Established prevention programs cannot be implemented in the vocational school setting without adaptation, as this setting differs from the normal school setting due to the combination of practical training in a company and vocational education at school. Obstacles to implementation might be limited class cohesion, time, and teacher involvement (see reference [9] for a detailed description of the vocational school setting in Germany).

### Mobile Interventions

Digital interventions and mobile technologies have the potential to overcome these barriers, especially with a vast majority of young people owning smartphones [10]. Mobile prevention programs offer a location- and time-independent as well as cost-effective way to deliver health-promoting content efficiently and in doses that can be easily integrated into daily life [11-13]. However, the participation rates (ie, the reach) of such mobile prevention programs vary [14-17]. Furthermore, in a recent review, Diestelkamp et al [18] report that despite growing evidence for the effectiveness of mobile substance use interventions, it is largely unclear which specific elements can be effectively implemented using mobile technology and how the extent to which individuals use this content may be associated with different outcomes.

Studies across different outcomes and delivery modes imply that multiple factors can influence the use and outcomes of digital interventions and that usage metrics vary across different types of interventions [19]. Previous research has shown that intensity of use (eg, number of log-ins) and active user engagement (eg, module completion) can influence intervention outcomes [19-21]. For example, a recent feasibility study investigating user engagement with a mobile smoke-free app showed a positive association between increased app use and cigarette abstinence [22]. Furthermore, a study focusing on a mobile intervention for heavy drinking and smoking among college students found that receiving more modules of the intervention was significantly associated with a lower likelihood of any drinking during the 14-day assessment period and a significant reduction in smoking at 1-month follow-up [23]. Thus, on a general level, usage patterns of digital interventions appear to play an important role in their effectiveness. However, it is still very unclear which specific usage metrics have an impact and how large the potential impact is, depending on the design of the intervention, its objectives, and its target population. It is also not clear how users’ background characteristics, such as sociodemographic factors, psychological characteristics, previous experiences with technologies, and the level of health symptoms, may influence intervention use and positive health outcomes.

### Meine Zeit ohne Intervention

In a recent randomized study [24,25], we transferred the school-based prevention approach of a voluntary commitment into an app-based program for vocational students (“Meine Zeit ohne” [MZo]; IFT-Nord Institut fuer Therapie- und Gesundheitsforschung GmbH). The app (see Multimedia Appendix 1 for app screenshots) was developed for implementation in vocational schools. In the trial, students in the intervention group (IG) received access to the MZo app, which was available for download at the Google Play Store and the Apple App Store (described in detail by Pietsch et al [25]). It includes an introduction to the overall theme of habits and on potential risky health behaviors concerning substance and media use as well as gambling. The app features a short explanation video demonstrating the use and goals of the app. Participants were invited to select 1 behavioral goal for the next 2 weeks: complete abstinence or substantial reduction regarding their use of cannabis, smoking (cigarettes or e-products), alcohol, digital social media use, gaming, gambling, or “another habit.” For the options of cannabis, alcohol, and gambling, only an abstinence goal could be selected. After choosing their behavioral goal and starting the challenge, participants received daily push notifications at fixed times (in the morning and at noon) to remind them to rate their subjective confidence in reaching their selected behavioral goal for the next 24 hours and to enter whether they succeeded in achieving their goal on the previous day. Following the last rating after 2 weeks, students could download and share a certificate regardless of their challenge results. The results suggest that this app-based intervention is feasible and effective in a vocational school setting [25]. Vocational students in the intervention arm classes were significantly (odds ratio [OR] 1.24, 95% CI 1.05-1.46; *P*=.01) more likely to report health-promoting changes such as reductions in substance use behavior and gambling as well as daily screen time for gaming or social media 30 days after the end of the app challenge.

As the effects on health-promoting change for a specific area were larger when students had chosen that particular challenge area, it seems that variations in *how* the MZo app was used (eg, the frequency of use and choice of challenge) and personal characteristics of users may have an impact on the intervention effects. In addition, as suggested by 2 recent studies, it may be interesting to explore whether the effects of a specific behavior reduction or abstinence are limited to that specific behavior or have positive (“spillover”) effects on other behaviors [26,27].

This paper presents an exploratory analysis, which focuses only on the IG and investigates who could be reached by the app and how the mobile intervention was used. The aims of this study were (1) to examine the characteristics of participants who used the app, (2) to explore the effectiveness of the mobile intervention depending on how the app was used and depending on individual participants’ characteristics, and (3) to study how variations in app use were related to participants’ baseline characteristics.

## Methods

### Ethical Considerations

Approval for the study was obtained from the ethics committee of the Center for Psychosocial Medicine at the University Medical Center Hamburg Eppendorf (approval number: LPEK-0121) and the responsible school authorities at each study site (Center for Education Monitoring and Quality Development at schools in Hamburg, Institut für Bildungsmonitoring und Qualitätsentwicklung; the Center for Prevention at the Institute for Quality Development at Schools in Schleswig-Holstein, Institut für Qualitätsentwicklung an Schulen Schleswig-Holstein; and the Bavarian State Ministry for Education and Cultural Affairs) prior to data collection. The study was conducted in accordance with the CONSORT (Consolidated Standards of Reporting Trials) guidelines and complied with the principles laid down in the Declaration of Helsinki [28]. It was registered in the German Clinical Trials Register public database (DRKS00023788), and a detailed study protocol was published [24]. Written informed consent was obtained from all participants prior to study enrollment. Participants did not receive any financial compensation for participating in the study. All substantial protocol deviations or modifications were communicated to the ethics committee and German Clinical Trials Register.

### Study Design and Participants

The MZo evaluation study was a 2-arm multicenter, cluster-randomized, waitlist-controlled trial (randomized controlled trial [RCT]) to test the effectiveness of a low-threshold, mobile app–based intervention in a sample of vocational students in Germany. The MZo app consisted of a 2-week intervention period followed by a 30-day follow-up period. Data were collected through web-based questionnaires and app use logs, starting in March 2021 and ending in April 2022. The RCT was conducted in 3 study centers in Germany—Munich, Kiel, and Hamburg.

At these sites, schools were consecutively recruited with the support of local school authorities or directly using digital and printed information materials, school conferences, etc. After initial agreement from school principalities, research staff, social school workers, or principals contacted the teachers at the participating school and informed them about the study’s aims and procedures. In the participating vocational schools, teachers and school principals selected classes to participate in the study. Participation in the challenge and the survey was voluntary. Since the study was conducted during the COVID-19 pandemic, enrollment, data assessment, and the introduction of the app could not take place in the classroom as initially planned but were done on the web if necessary. Randomization was done at the class level and performed before the baseline assessment. To achieve this aim, 2 classes were each paired into similar dyads based on the three class characteristics: (1) frequency of in-school education, (2) educational area (eg, technical or IT, services, and trade), and (3) year of training (eg, first, second, and third) [25]. Paired classes were then randomized into the IG or control group. Researchers had no access to the app data during the intervention.

### Measures

#### Overview

This analysis used baseline measurements, follow-up measures (all self-reported), and mobile app use log data from study participants in the IG. Study variables are described in detail below. Sociodemographic data were collected at baseline via web-based questionnaires and included sex or gender, age, migration status, income, and highest level of education.

App usage metrics were collected through usage log files recorded by the mobile phone app on an external server. Usage log files included, for each participating user, the registration code to match the app data with the data collected in the questionnaires, information about the challenge (eg, challenge choice, decision to choose a reduction or abstinence goal, and reduction goal in units or time), confidence, as well as success ratings in meeting their challenge goal for each day. The log files were retrieved from the server at the end of the intervention period. Usage metrics could be divided into 3 categories, which are summarized in Textbox 1. The definitions of the variables frequent use and congruent use are described in detail below.

**Description of usage metrics.**

- Selected challenge: Users selecting a specific challenge (cannabis, smoking or vaping, alcohol, gaming, social media, gambling, and “other”).
- Frequent use: Users using the app frequently.
- Congruent use: Users selecting a challenge congruent to his or her personal relevance.

#### Frequent Use

The number of days of use was determined by the total number of days on which participants made a confidence rating entry in the app. The confidence rating variable was chosen because it had to be rated on the respective day. Thus, this variable determined whether or not participants were actively using the app on a certain day during the challenge. As the number of days of active use showed a left-skewed distribution, further analysis was done using the binary variable frequent use based on a median split (eg, above or below 4 days of active use). Thus, nonfrequent use was defined when users actively used the app for up to 4 days, and frequent use was defined when users actively used the app for more than 4 days.

#### Congruent Use

The congruent use variable identified users who selected a challenge that met their personal relevance. Personal relevance was defined for each behavioral area (tobacco, e-cigarettes, alcohol, cannabis, gambling, gaming, and digital media) when a predefined threshold for the frequency of that behavior was exceeded at baseline assessment. For cannabis use, personal relevance was defined as a positive 30-day prevalence. For alcohol, cigarettes, and e-products, personal relevance was defined as consumption at least once a week (ie, on more than 4 days) in the past 30 days. For social media, gaming, and gambling, personal relevance was defined using the median split. Thus, for social media, personal relevance was determined if social media use exceeded 4 hours per day, and for gaming, if gaming days per month exceeded 3 days. For gambling, personal relevance was defined as at least 1 gambling day per month. Congruent use was determined when personal relevance for a particular substance-related behavior or behavioral addictive–related behavior was met and a participant actually chose a challenge for the same behavior. For students without a personally relevant behavior (ie, no behavior exceeding these thresholds), congruent use was set when the challenge “other” was selected. For example, congruent use was set when someone who smoked more than 4 days a month selected the smoking challenge.

#### Personal Relevance Score

To further explore how the level of personal risk regarding substance-related and addictive behavior influenced health-promoting change, a continuous variable named personal relevance score was created based on the personal relevance variables for tobacco, e-cigarettes, alcohol, cannabis, gambling, and gaming as well as digital media (for definitions of thresholds, see the *Congruent Use* section). The intention was to quantify the number of personal relevant habits. The personal relevance score was the sum of personal relevant behaviors for each participant; for example, when a participant smoked cigarettes at least once a week and drank alcohol at least once a week, his or her personal relevance score was set to 2. The values of the personal relevance score ranged between 0 (no area of personal relevance) and 7 (7 areas of personal relevance).

#### Outcome Variables: Health-Promoting Change Variables

For this analysis, the dichotomous health-promoting change variables and a variable called general adverse health behavior improvement (GAHBI) were created according to Pietsch et al [25]. For alcohol, gambling, and cannabis use, a health-promoting change was defined as a positive 30-day prevalence at baseline compared with a zero 30-day prevalence at follow-up. For cigarettes and e-products, change was defined as a reduction of at least 50% in the monthly number of cigarettes or e-product units from baseline to follow-up. For social media and gaming, change was defined as a reduction of at least 20 minutes in daily screen time. The GAHBI was coded as health-promoting change if there was at least 1 change in the abovementioned areas.

### Statistical Analysis

**Figure 1:**
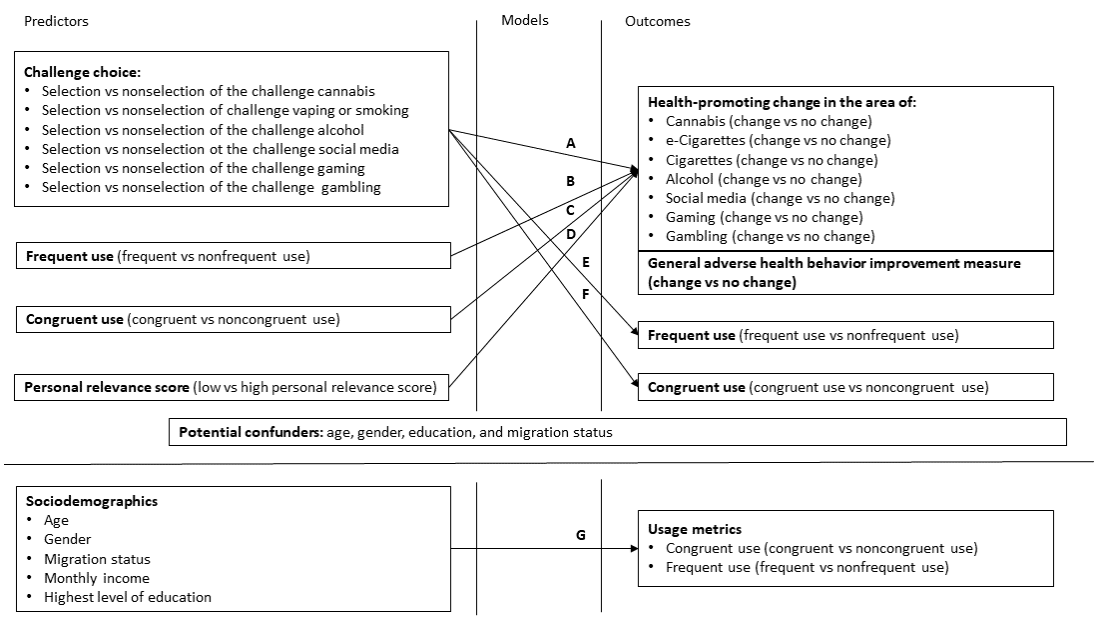
Main analyses.

Statistical analysis was performed using SAS for Windows (version 9.4 TS Level 1M7; SAS Institute Inc). Statistical significance was set at *P*≤.05 (but exact *P* values are reported). Descriptive statistics were calculated using SPSS Statistics Release (version 26; IBM Corp). Differences in baseline characteristics between app users and nonapp users were assessed using Bonferroni-corrected 2-tailed *t* tests and chi-square tests. The relationship between the predictors (challenge choice, frequent use, congruent use, and personal relevance score) and the outcome variables (change in all dichotomous health-promoting change variables as well as the GAHBI [change vs no change]) was analyzed using binary multilevel mixed-effects logistic regression models (SAS Glimmix), accounting for the clustered structure of the data with clustering occurring within federal states, schools, and classes (see models A-D in Figure 1). For these results, ORs with corresponding 95% CIs are reported. Age, sex or gender, education, and migration status as potential confounders were included as fixed effects to the models. For the models for alcohol, cannabis, and gambling, the number of included confounders had to be reduced in order to achieve convergence. For the models for alcohol and cannabis, age and migration status were added, and for the model for gambling, migration status only.

The relationship between the usage metric of congruent use (yes vs no) and the selected challenge as well as frequent use was analyzed using binary multilevel mixed-effects logistic regression models (see models E and F in Figure 1). This was also done to analyze the relationship between the usage metric of frequent use and the selected challenge as well as congruent use. ORs, 95% CI, and *P* values were calculated and reported for all outcomes. As the usage metrics were important variables in this study, they were further tested for associations with the sociodemographic baseline variables age, gender, migration status, monthly income, and highest level of education using multilevel mixed-effects logistic regression models (see model G in Figure 1).

## Results

### Overview

**Figure 2:**
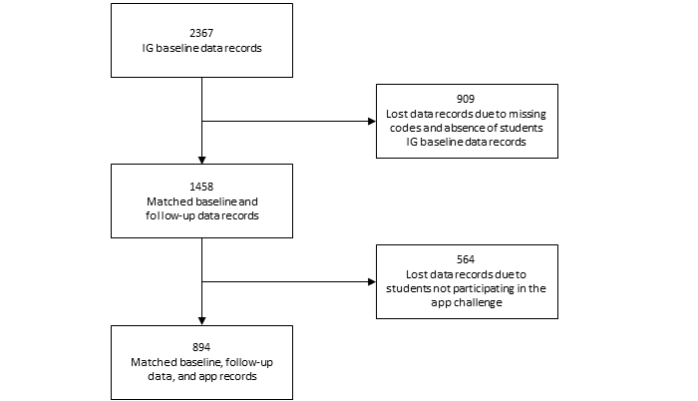
Participant flowchart. IG: intervention group.

Altogether, 2367 (51.6%) of 4591 students were randomized to the IG. The baseline and follow-up data of 1458 (61.6%) of the 2367 students in the IG could be matched by the corresponding codes of participants in the baseline and follow-up assessments. A total of 909 students were lost to follow-up due to absence or missing survey codes. Of these 1458 students, 894 (61.3%) started a challenge. Thus, 894 data records with baseline, follow-up, and app data could be included in the analysis (Figure 2).

### Sample Characteristics

The included sample (n=2367) consisted of 1308 (55.3%) male participants, 1040 (43.9%) female participants, and 19 (0.8%) participants who did not identify as binary. Their mean age at baseline was 19.2 (SD 4.2) years, and 786 (33.2%) reported a migrant background. Reported 30-day prevalence rates were 817 (34.5%) for smoking, 1536 (64.9%) for alcohol use, 294 (12.4%) for gambling, and 379 (16%) for cannabis use. In addition, IG students used social media for an average of 222.2 (SD 185.5) minutes per day and reported playing video games for an average of 99.6 (SD 140.1) minutes per day. The analysis revealed several differences between app users (students who used the app at least 1 time) and nonapp users (students who did not use the app at all) at the sociodemographic level. Students who used the app on average had a younger age and were more likely to have no migrant background, to have a medium monthly income, to have a middle school diploma, and to have vocational training in commerce, industry, and technology (Table 1).

**Table 1:** Description of the overall IG^a^ sample, the IG sample with matched baseline and follow-up data records, and differences between app users and nonapp users in the matched IG sample assessed with Bonferroni-corrected 2-tailed t tests and chi-square tests.

Sociodemographics		Overall IG (n=2367)	Matched IG (n=1458)	App users (n=894)	Nonapp users (n=564)	*P* value
Age (years), mean (SD)		19.2 (4.2)	19.0 (3.5)	18.7 (3.5)	19.4 (3.6)	<.001
**Sex, n (%)**						
	Male	1308 (55.3)	830 (56.9)	525 (58.7)	305 (54.9)	—^b^
	Female	1040 (43.9)	618 (42.4)	363 (40.6)	255 (45.2)	.22
	Other	19 (0.8)	10 (0.7)	6 (0.7)	4 (0.7)	—
**Migration background, n (%)**						
	No	1581 (66.8)	1053 (72.2)	673 (75.3)	380 (67.4)	—
	Yes	786 (33.2)	405 (27.8)	221 (24.7)	184 (32.6)	.001
**Monthly income (€)^c^, n (%)**						
	>1000	345 (14.6)	189 (13)	120 (13.4)	69 (12.2)	—
	600-999	1184 (50)	790 (54.2)	506 (56.6)	284 (50.4)	.01
	<600	838 (35.4)	479 (32.9)	268 (30)	211 (37.4)	—
**Highest education, n (%)**						
	High school diploma	473 (20)	292 (20)	154 (17.2)	138 (24.5)	—
	Middle school diploma	1247 (52.7)	837 (57.4)	578 (64.7)	259 (45.9)	<.001
	Below middle school diploma	647 (27.3)	329 (22.6)	162 (18.1)	167 (29.6)	—
**Educational area, n (%)**						
	Vocational preparation	120 (5.1)	46 (3.2)	25 (2.8)	21 (3.7)	—
	Commerce, industry, and technology	1307 (55.2)	886 (60.8)	608 (68)	278 (49.3)	—
	Economics and management	554 (23.4)	290 (19.9)	127 (14.2)	163 (28.9)	<.001
	General school-based education	386 (16.3)	236 (16.2)	134 (15)	102 (18.1)	—
Number of personal relevant behaviors, mean (SD)		2.11 (1.4)	2.07 (1.3)	2.08 (1.3)	2.06 (1.3)	.74

^a^IG: intervention group.

^b^Not applicable.

^c^As of June 12, 2024, a currency exchange rate of €1=US $1.075 is applicable.

### Usage Metrics and Personal Relevance Score

A total of 894 (61.3%) of the 1458 students in the IG sample with matched data started the challenge (Figure 2). The mean number of days of active use was 5.62 (SD 4.76). With up to 4 days of active use, 417 (46.6%) of the 894 students using the app were nonfrequent users, 466 (52.1%) were frequent users with more than 4 days of active use, and 11 (1.2%) selected a challenge but had no days of active use. A total of 85 (9.5%) actively used the app throughout the entire intervention period of 14 days.

The challenge area most often chosen in the matched sample (n=894) was social media (n=332, 37.1%), followed by “other challenge” (n=159, 17.8%), alcohol (n=128, 14.3%), smoking or vaping (n=127, 14.2%), gaming (n=79, 8.8%), and cannabis (n=38, 4.3%). The least chosen challenge was gambling with 31 (4.2%) students. A total of 407 (45.5%) students chose a challenge congruent with their personal relevance. Of these 407 students, 126 (31%) selected a challenge in the category social media, 84 (20.6%) in the category smoking or vaping, 79 (19.4%) in the category alcohol, 61 (15%) in the category gaming, 23 (5.7%) in the category cannabis, 19 (4.7%) in the category “other challenge,” and 15 (3.7%) in the category gambling.

The mean number of personal relevant behaviors was 1.88 (SD 1.32). Of all 894 students, 296 (33.1%) had a personal relevance score of 1, followed by 245 (27.4%) with a personal relevance score of 2, 245 (27.4%) with a personal relevance score above 2, and 108 (12.1%) with a personal relevance score of 0.

### Association Between Challenge Choice and Health-Promoting Change or Congruent Use

ORs for health-promoting change in some areas were associated with the choice of challenge, particularly when the area of the challenge and health-promoting change were the same (eg, smoking or vaping challenge and health-promoting change in smoking; see model A in Figure 1). The results are displayed in Table 2. The ORs for health-promoting change increased for cigarettes (OR 3.51, 95% CI 2.14-5.76; *P*<.001) and e-cigarettes (OR 2.87, 95% CI 1.69-4.85; *P*<.001) for users who chose the smoking or vaping challenge. For users choosing the social media challenge, the ORs for a health-promoting change for social media increased (OR 1.66, 95% CI 1.25-2.20; *P*=.001) but decreased for a health-promoting change for smoking (cigarettes: OR 0.55, 95% CI 0.34-0.89; *P*=.001 and e-cigarettes: OR 0.56, 95% CI 0.33-0.92; *P*=.02). ORs for health-promoting change also increased for gaming (OR 1.77, 95% CI 1.09-2.89; *P*=.02) for users who chose the respective challenge. For users who chose the challenge “other challenge,” ORs for health-promoting change increased for alcohol (OR 2.15, 95% CI 1.13-4.09; *P*=.02) but decreased for GAHBI (OR 0.63, 95% CI 0.42-0.96; *P*=.03) and social media (OR 0.66, 95% CI 0.45-0.97; *P*=.03). ORs were not reported when the number of participants with a health-promoting change for a particular model was less than 5.

Furthermore, the likelihood for the choice of a congruent challenge (see model F in Figure 1) was higher for users who chose the challenges smoking or vaping (OR 2.74, 95% CI 1.80-4.16; *P*<.001), alcohol (OR 2.17, 95% CI 1.45-3.26; *P*<.001), and gaming (OR 4.57, 95% CI 2.58-8.10; *P*<.001) and lower for users who chose the challenges social media (OR 0.57, 95% CI 0.34-0.77; *P*=.001) and “other challenge” (OR 0.10, 95% CI 0.06-0.17; *P*<.001).

**Table 2:** ORs^a^ with a 95% CI for health-promoting change for students who chose a challenge corresponding to the health-promoting change area (see model A in Figure 1).

Health-promoting change	Participants, n	Events, n	OR (95% CI)	*P* value
Cigarettes	894	32	3.51 (2.14-5.76)	<.001
e-Cigarettes	894	26	2.87 (1.69-4.85)	<.001
Social media	894	185	1.66 (1.25-2.20)	<.001
Gaming	894	34	1.77 (1.09-2.89)	.02
Alcohol	894	9	1.08 (0.53-2.21)	.84
Cannabis	—^b^	—	—	—
Gambling	—	—	—	—

^a^OR: odds ratio.

^b^Not available.

### Association Between Congruent Use or Frequent Use and Health-Promoting Change Variables

**Figure 3:**
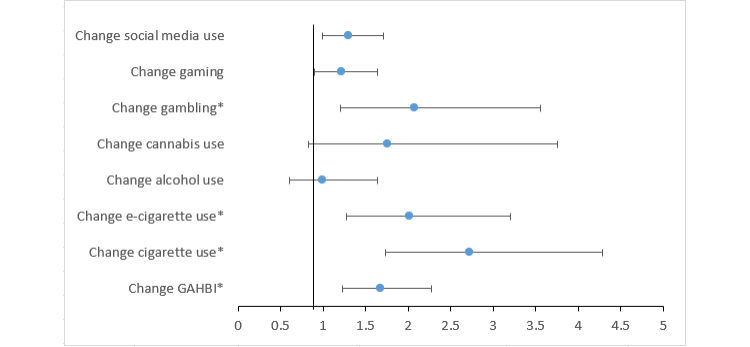
Health-promoting change for students choosing a congruent challenge compared to students choosing an incongruent challenge (adjusted odds ratios with 95% CI). GAHBI: general adverse health behavior improvement. *P<.05.

Congruent use was associated with increased odds of health-promoting change (see model C in Figure 1) for cigarettes (OR 2.72, 95% CI 1.73-4.29; *P*<.001), e-cigarettes (OR 2.02, 95% CI 1.28-3.20; *P*=.003), gambling (OR 2.07, 95% CI 1.21-3.55; *P*=.008), and GAHBI (OR 1.67, 95% CI 1.23-2.27; *P*=.001). Thus, students with a personal relevance score for cigarettes who chose cigarettes as their challenge (congruent use) were 2.72 times more likely to report a health-promoting change in cigarettes than students with a personal relevance score for cigarettes who did not choose smoking but another challenge (incongruent use). The results are displayed in Figure 3. Frequent use showed lower odds for health-promoting change (see model B in Figure 1) for gambling (OR 0.47, 95% CI 0.26-0.86; *P*=.009; Multimedia Appendix 2).

### Association Between the Personal Relevance Score and Health-Promoting Change

The odds for health-promoting change increased with each additional unit of the personal relevance score (see model D in Figure 1) except for alcohol (Table 3). Thus, there was an increase in the odds for health-promoting change between personal relevance scores of 0 and 1 and a further increase in odds between personal relevance scores of 1 and 2, meaning that those with a personal relevance score of 7 (personal relevance score for alcohol, cigarettes, e-cigarettes, cannabis, social media, gaming, and gambling) were most likely to show a health-promoting change. To sum it up, the more problematic consumption habits a student had, the more likely he or she was to have a health-promoting change, except for a health-promoting change for alcohol.

**Table 3:** ORs^a^ with a 95% CI for health-promoting change increasing with each additional unit of the personal relevance score, except for alcohol.

Health-promoting change	Participants, n	Events, n	OR (95% CI)	*P* value
Cigarettes	894	99	1.63 (1.40-1.92)	<.001
e-Cigarettes	894	90	1.73 (1.47-2.04)	<.001
Social media	894	429	1.27 (1.14-1.41)	<.001
Gaming	894	248	1.25 (1.12-1.41)	<.001
Alcohol	894	71	0.97 (0.80-1.17)	.72
Cannabis	894	30	1.93 (1.49-2.50)	<.001
Gambling	894	63	2.43 (1.99-2.98)	<.001
GAHBI^b^	894	649	1.69 (1.46-1.95)	<.001

^a^OR: odds ratio.

^b^GAHBI: general adverse health behavior improvement.

### Associations Between Congruent Use and Sociodemographic Variables

Congruent use was found to be a relevant predictor of health-promoting changes and was therefore analyzed for associations with participants’ sociodemographic characteristics such as age, gender, income, migration, and highest level of education. With the exception of migrant background, none of the included socioeconomic variables were found to be associated with congruent use. The odds for congruent use were lower for students with a migrant background compared to students with no migrant background (OR 0.67, 95% CI 0.49-0.94; *P*=.02).

## Discussion

### Principal Findings

The aim of this study was to examine the reach of a low-threshold mobile prevention program and its effectiveness depending on participant characteristics and actual app use. To achieve this aim, metrics describing the use of the mobile intervention and a relevance score related to substance- and addiction-related personal risk and their associations with health-promoting change were analyzed.

The exploratory study revealed three main findings: (1) approximately 6 out of 10 students (894/1458, 61.3%) used the app and were thus reached by the prevention intervention; (2) almost half (407/894, 45.5%) of the app users used the app in congruence with personal relevance, suggesting a relatively high awareness of their personal habitual behaviors; and (3) as health-promoting changes were influenced by congruent use, choice of challenge, and personal relevance score, it can be argued that these associations provide empirical support for differential effectiveness of the app.

The proactive invitation to participate in the program and in the study in vocational school classes, combined with the offer of a low-threshold mobile intervention, reached almost 2 out of 3 students (894/1458, 61.3%) in the matched IG sample who downloaded the MZo app and started a challenge. This participation rate appears to be substantial, given the fact that the MZo program was not integrated into regular school lessons, but students were encouraged to download the app to their personal smartphones. Moreover, the introduction of the program was rather brief, and about one-quarter of the students were introduced to the program on the web. Nevertheless, the participation rate matches other mobile phone intervention programs with similar target groups in Switzerland and Germany, focusing on reducing problematic drinking or supporting smoking cessation, which attracted between 50% and 70% of the students [14-17,29]. However, a recent review suggests that local and personal recruitment of users leads to higher adherence or program participation [30]. Due to limited access to schools during the COVID-19 pandemic, 24.1% (1106/4591) of the students were recruited on the web. A higher rate of students might have been reached if recruitment had entirely been done during face-to-face school visits. In terms of who used the app, the results showed that app users were on average younger and more likely to have no migrant background; to have a medium monthly income; and to have a vocational training in commerce, industry, and technology. Furthermore, there was an association between congruent use of the app and no migrant background.

Results indicate that frequent use had no influence on health-promoting change. It could be argued that for this low-threshold mobile app, active use was not a necessary requirement for health-promoting change, as users received reminders to continue the challenge twice a day, and no further content about the challenge or the goal achievement was provided by the app once the challenge was started. This distinguishes the app from other mobile prevention apps [22,31].

Results also indicate that the odds for health-promoting behavior change were higher when the area of individual challenge choice was equal to the area of behavior change, challenge choice was congruent with a behavior of personal relevance (eg, a regular smoker chose the smoking challenge), and the individual risk of engaging in addictive behaviors was high. Congruent use almost tripled the odds for a health-promoting change for cigarettes and doubled the odds for a health-promoting change for e-cigarettes and gambling. Moreover, congruent use was the only variable in the analysis that provided an increase in the odds for general adverse health behavior improvement. This effect was based on the health-promoting changes in cigarettes and e-cigarettes.

As students’ challenge choices were self-selected, these results indicate that a certain awareness of problematic habitual behaviors in combination with a motivation to change these behaviors may lead to health-promoting changes. Recent studies confirm that awareness and intrinsic motivation are important factors for behavioral change [32,33]. However, in contrast to Brailovskaia et al [26,27], the effects of a specific behavior reduction or abstinence were limited to the corresponding health-promoting change area. Thus, a “spillover effect” from a challenge in one behavioral domain did not generalize to other outcomes in this study. The results also suggest that it is more difficult to achieve the goal of a health-promoting change when it is defined as complete abstinence from a substance (eg, alcohol and cannabis). One reason for this may be that the app was designed as a low-threshold intervention targeting habitual behaviors rather than an intervention providing therapeutic support to change behavior. In this study, 1 in 3 adolescents reported to drink alcohol regularly [4]. Therefore, total alcohol abstinence might be an unrealistic goal for this group. The same may be true for cannabis, especially for the 8.6% of the 18- to 25-year-old adults who reported to consume it regularly [4].

Furthermore, health-promoting change for social media and gaming, although defined as reduction goals, seems to be more difficult to achieve than the reduction of cigarettes or e-cigarettes. One reason for this may be that the data collection for this study took place during the COVID-19 pandemic. Limited real-life contact with peers due to COVID-19 restrictions led to an increased frequency and duration of digital media use [34]. Thus, it could be argued that reducing gaming and social media time may have been more challenging during the COVID-19 pandemic because there were fewer social contact alternatives.

The results also show that a challenge choice increased the odds for the respective health-promoting change areas for cigarettes, e-cigarettes, social media, and gaming, except for alcohol. This can be seen as an indicator that the app effectively supported users who were willing to change a particular behavior. These results also confirm that reducing consumption is easier to achieve than total abstinence (eg, alcohol). The effectiveness of the app is further demonstrated by the fact that the personal relevance score was associated with increased odds for health-promoting change for cigarettes, e-cigarettes, cannabis, gambling, gaming, and social media. It is likely that students with a higher degree of problematic health behavior as indicated by a high relevance score were more aware of their behavior and thus more motivated to change. On the other hand, it could also be expected that these users would be less likely to achieve a health-promoting change with such a minimalistic support program, as these users are likely to have greater problems with controlling addictive behaviors. It would be informative to know more about the motivational background of the users in more detail. However, this requires qualitative data, for example, in the context of mixed method studies, which appears useful when testing new complex interventions.

### Limitations

This study has a number of limitations: (1) the attrition rate was high and biased (1473/2367, 62.2%), partly due to the anonymization procedure, which allowed linking of baseline, follow-up, and app data without collecting any personal information. (2) As all data were based on self-reported data, social desirability and a recall bias may have influenced the results of the study. (3) Data collection for the study took place during the COVID-19 pandemic in 2021-2022, with varying restrictions in schools concerning data collection and social contact. These restrictions also affected the lives, substance use, and other behaviors of the students and thus the results of the study and their generalizability. (4) The number of health-promoting change events for alcohol, cannabis, and gambling was low, so these results should be interpreted with caution. (5) The results concerning the personal relevance score should also be interpreted with caution. As the formal risk screeners for addictive behaviors did not show relevant results, the personal relevance score was pragmatically created with the aim of showing habitual behavior. (6) As a convenience sample was recruited from school classes and students participated voluntarily, the results cannot be generalized to vocational school students in Germany.

### Comparisons With Prior Work

The main finding of this study, the influence of congruent use and challenge choice on the intervention outcomes, partially confirms previous findings of Brailovskaia et al [26,27]. On the one hand, health promotion can be achieved through behavioral abstinence or reduction, but on the other hand, in contrast to Brailovskaia et al [26,27], the results of this study indicate that the effects of a specific behavior reduction or abstinence are limited to that specific behavior. The odds of health-promoting change increased either when students chose a challenge corresponding to their personal relevance or when the challenge choice met the respective area of health-promoting change. However, it was not investigated whether there were other positive health effects, for example, on physical activity or on mental health. Future research should further deepen the knowledge of the effects of voluntary commitment on health-related outcomes. The finding that congruent use and individual choice can play an important role in the effectiveness of the MZo intervention supplements previous research discussing how the most appropriate metrics of use may differ between different types and aims of interventions and how the exploration of use can help to understand which metrics are most associated with effectiveness [19,22,31]. In addition, the results of the study point toward harm or risk reduction rather than abstinence-related prevention for adolescents. Furthermore, there was an influence of the personal relevance score on health-promoting change. Future research should therefore focus on an in-depth exploration of the most appropriate measures of use of different interventions, on how these mechanisms of use influence the effectiveness of different interventions, and on how these mechanisms are influenced by intervention design and personal characteristics. Looking at the role of habitual behavior in the effectiveness of the app could be another area for future research.

To improve future versions of the MZo program, the reach of the program could be extended by embedding it in regular school curricula. This may increase awareness of the MZo program but would also require higher teacher involvement. Given the current results, congruent use of the app should be more strongly encouraged to increase the effectiveness of the program. Moreover, individual suitability may be increased by providing additional individual options, such as reduction goals in the areas of gambling, alcohol, and cannabis.

### Conclusions

The MZo program appears to be promising in supporting behavioral change across a spectrum of addictive behaviors. The prevention approach based on voluntary commitment can be introduced to vocational students with minimal effort and offers autonomous and mobile use with a low threshold for participating schools and students. It has the potential to reach and raise awareness among adolescents before habits become addictions.

## Appendix

Multimedia Appendix 1 Screenshots of the Meine Zeit ohne app.

Multimedia Appendix 2 Odds ratio with 95% CI for health-promoting change for high-use intensity.

## Abbreviations

CONSORT: Consolidated Standards of Reporting Trials
GAHBI: general adverse health behavior improvement
IG: intervention group
MZo: Meine Zeit ohne
OR: odds ratio
RCT: randomized controlled trial
