# Supplementary figures and images for: Mobile App Intervention to Reduce Substance Use, Gambling, and Digital Media Use in Vocational School Students: Exploratory Analysis of the Intervention Arm of a Randomized Controlled Trial
Source: JMIR Mhealth Uhealth. 2024 Jul 23;12:e51307. doi: 10.2196/51307 (PMC11303885; doi:10.2196/51307)

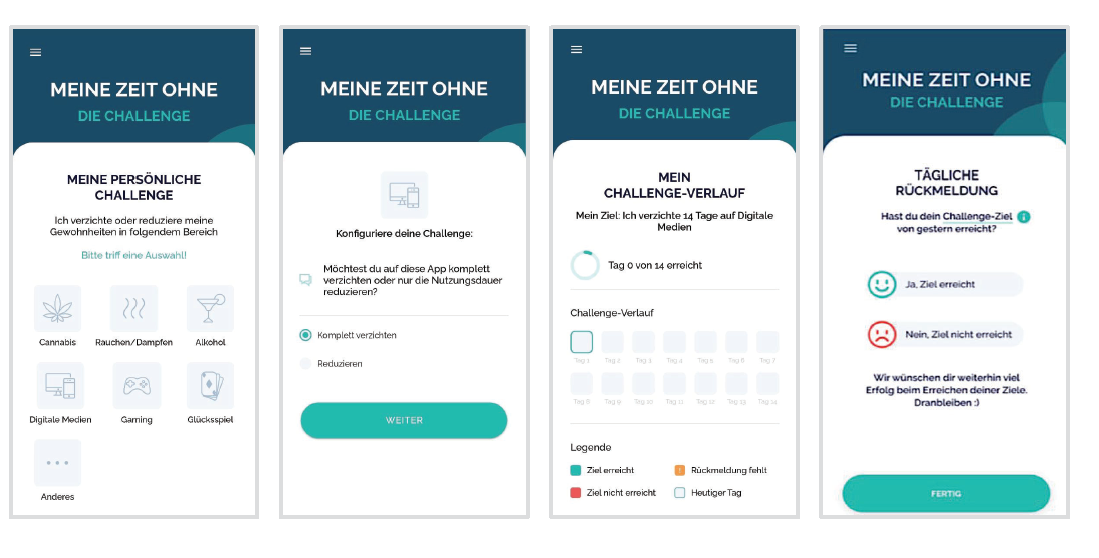

Supplement: Multimedia Appendix 1 [file mhealth_v12i1e51307_app1.png]
