# Supplementary material for: Mobile App Intervention to Reduce Substance Use, Gambling, and Digital Media Use in Vocational School Students: Exploratory Analysis of the Intervention Arm of a Randomized Controlled Trial
Source: JMIR Mhealth Uhealth. 2024 Jul 23;12:e51307. doi: 10.2196/51307 (PMC11303885; doi:10.2196/51307)
Supplement: Multimedia Appendix 2 [file mhealth_v12i1e51307_app2.docx]

| **Health promoting change for** | n | Events | OR | 95% CI | p-value |
| --- | --- | --- | --- | --- | --- |
| Cigarettes | 894 | 99 | 0.67 | 0.43 – 1.04 | 0.073 |
| E-Cigarettes | 894 | 90 | 0.75 | 0.47– 1.19 | 0.219 |
| Social Media | 894 | 429 | 1.25 | 0.95– 1.63 | 0.114 |
| Gaming | 894 | 248 | 0.85 | 0.63 – 1.16 | 0.300 |
| Alcohol | 894 | 71 | 1.04 | 0.63 – 1.72 | 0.885 |
| Cannabis | 894 | 30 | 0.41 | 0.19– 1.00 | 0.049 |
| Gambling | 894 | 63 | 0.47 | 0.26 – 0.83 | 0.009 |
| GAHBI^a^ | 894 | 649 | 0.82 | 0.61 – 1.11 | 0.196 |

^a^= General Adverse Health Behavior Variable
